# Supplementary material for: Identification of photoperiod-induced specific miRNAs in the adrenal glands of Sunite sheep (Ovis aries)
Source: Front Vet Sci. 2022 Jul 22;9:888207. doi: 10.3389/fvets.2022.888207 (PMC9354845; doi:10.3389/fvets.2022.888207)
Supplement: Supplementary file 1 [file Data_Sheet_1.ZIP › Supplementary file/miRDeep2 file.zip!/miRDeep2 file/Novel_Structure/SPLP42A1/Novel_38.pdf]

Provisional ID	:	NC_019460.2_6774
Score total	:	2.4
Score for star read(s)	:	-1.3
Score for read counts	:	0
Score for mfe	:	2.1
Score for randfold	:	1.6
Score for cons. seed	:	
Total read count	:	682
Mature read count	:	678
Loop read count	:	0
Star read count	:	4

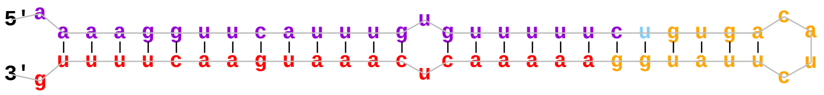

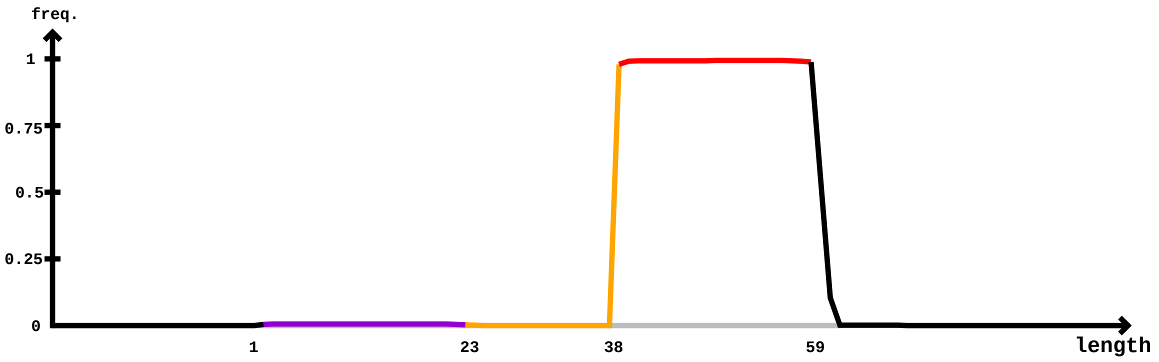

Star Mature

```
5'- gaaauaauacuggguuggccaaaagguucauuuguguuuuucugugacaucuuauggaaaaacucaaaugaacuuuugggccaacccaaugaaaagcuccaaucccuuca  -3'   obs
    gaaauaauacuggguuggccaaaagguucauuuguguuuuucugugacaucuuauggaaaaacucaaaugaacuuuugggccaacccaaugaaaagcuccaaucccuuca         exp
    ..........((((((((((.(((((((((((((.((((((((((((....)))))))))))).))))))))))))).))))))))))......................   reads     mm      sample
    ....................aaaagguucauuuguguuuu......................................................................   1         0       seq
    ....................aaaagguucauuuguguuuuu.....................................................................   1         0       seq
    ....................aaaagguucauuuguguuuuuc....................................................................   1         0       seq
    .....................aaagguucauuugGguuuuucu...................................................................   1         1       seq
    .........................................................aaaaacucaaaugaacuu...................................   1         0       seq
    .........................................................aaaaacucaaaugaacuuuC.................................   1         1       seq
    .........................................................aaaaacucaaaugaacuuuA.................................   1         1       seq
    .........................................................aaaaacucaaaugaacuuuuA................................   42        1       seq
    .........................................................aaaaaAucaaaugaacuuuug................................   1         1       seq
    .........................................................aaaaacucaaaugaacCuuug................................   2         1       seq
    .........................................................aGaaacucaaaugaacuuuug................................   1         1       seq
    .........................................................aaaaacucaaaugaacuuuuU................................   1         1       seq
    .........................................................aaaaacucaGaugaacuuuug................................   2         1       seq
    .........................................................aaaaacucaaaugaacuuuug................................   446       0       seq
    .........................................................Gaaaacucaaaugaacuuuug................................   4         1       seq
    .........................................................aaaaacucaaaugaacuuuugg...............................   91        0       seq
    .........................................................aaaaacucaaaugaacuuuuUg...............................   8         1       seq
    .........................................................aaaaacucaaaugaacuuuuCg...............................   1         1       seq
    .........................................................aaaaacucaaaugaacuuuuggU..............................   54        1       seq
    .........................................................aaaaacucaaaugaacuuuuggg..............................   13        0       seq
    ..........................................................aaaacucaaaugaacuuu..................................   1         0       seq
    ..........................................................aaaacucaaaugaacuuuug................................   2         0       seq
    ..........................................................aaaacucaaaugaacuuuuUg...............................   3         1       seq
    ..........................................................aaaacucaaaugaacuuuuggU..............................   2         1       seq
    ...........................................................aaacucaaaugaacuuuuggA..............................   1         1       seq
    ...................................................................augaacuuuugggccaaUcc.......................   1         1       seq
```
